# Is migration in later life good for wellbeing? A longitudinal study of ageing and selectivity of internal migration

**DOI:** 10.1111/area.12428

**Published:** 2018-05-17

**Authors:** Nissa Finney, Alan Marshall

**Affiliations:** ^1^ School of Geography and Sustainable Development University of St Andrews St Andrews Fife UK; ^2^ School of Social and Political Science University of Edinburgh Edinburgh UK

**Keywords:** England, English Longitudinal Study of Ageing, internal migration, later life, selective migration, wellbeing

## Abstract

Migration scholarship has recently paid attention to lifecourse and non‐economic effects of moving house. Yet consideration of the effects of internal migration in later life has been relatively neglected despite their implications for social and spatial inequalities. Thus we address two questions: how trajectories of wellbeing in later life vary for movers and non‐movers, and how the event of moving affects wellbeing. In both cases we distinguish between “voluntary” and “involuntary” movers. We use 10 years (2002–2012) of the English Longitudinal Study of Ageing (ELSA) to analyse trends in wellbeing for age cohorts and to examine how wellbeing changes through the event of moving. The Control, Autonomy, Selfrealisation and Pleasure (CASP‐19) measure of wellbeing is used. We find that, after controls for demographic and socio‐economic characteristics, involuntary movers have lower levels of wellbeing than stayers or voluntary movers; and involuntary movers experience a stabilisation in the decline in wellbeing following migration which is not seen for voluntary movers. So, migration in later life is good for wellbeing, maintaining advantageous wellbeing trajectories for voluntary movers and improving wellbeing trajectories for involuntary movers. These findings imply a rich potential of ELSA and similar longitudinal datasets for examining residential mobility; the need for ageing inequalities studies to take more account of residential mobility; the need for internal migration scholarship to pay greater attention to reason for move; and for policy to consider the potentially beneficial effects of residential mobility in later life, particularly for those in adverse circumstances.

## 1 INTRODUCTION: LATER LIFE, MIGRATION AND WELLBEING

This paper engages with a field that has been relatively neglected by Geography and wider Social Science scholarship: the effects of internal migration in later life. In migration literature, recent attention to lifecourse and longitudinal approaches has focused on young adults and working ages (Coulter et al., 2016; Finney, 2011), for understandable reasons of the high migration levels in these life stages. There has also been a growth in understandings of the internal migration of children within the broader context of family migration studies and Children's Geographies (e.g., Cooke, 2008; Yarwood & Tyrrell, 2012). Although some important work on internal migration in later life exists, this is a relatively neglected life phase, despite recognition of specificities of late‐life migration (Rees & Hardill, 2015; Warnes, 1992) and high levels of desire to move among older people (Hillcoat‐Nalletamby & Ogg, 2014). Evandrou et al. (2010) provide a valuable account of the factors associated with migration in later life, pointing to the importance of changes in partnership status, health and economic activity as drivers of migration. They argue for a lifecourse perspective to understand how moves are triggered by particular events, such as retirement or widowhood.

In addressing the question of the effects of internal migration on wellbeing in later life, we offer three original contributions, substantive and methodological. The first is the empirical combination of analysis of later life, wellbeing and internal migration, to date neglected; the second is the examination of how the effect of migration on wellbeing in later life varies according to reason for move, a rare approach in quantitative work; and the third is the use for these purposes of the English Longitudinal Study of Ageing (ELSA) and longitudinal analyses. These contributions augment understanding of the selectivity of migration in later life and the potential of quantitative analysis to complement the existing body of work by evidencing processes of migration selectivity and their effect for the older population as a whole.

The embracing of lifecourse approaches in migration literature has been accompanied by increased attention to the effects of migration beyond the economic and material (Nowok et al., 2013). Particularly notable in this regard is work on health encompassing both physical and mental health, including wellbeing. It is in the health arena that the ageing literature has most directly grappled with internal migration, with particular attention given to migration experiences of frail older adults in relation to care, both international (Hardill et al., 2005) and internal (Robards et al., 2014). Indeed, ageing literature has tended to address these questions from the perspective of living arrangements, encompassing moves to care institutions (see Feng et al., 2017; for example). Internal migration in later life is, however, more diverse in its drivers, how it is experienced and how it is selective than this body of work implies. This paper views internal migration as a process of social and spatial inequalities, both in its drivers and consequences (Smith et al., 2015), and aims to augment understanding of the significance for wellbeing of being able to move and of the reason for moving.

Nowok et al. (2013) provide an excellent platform for this study in their analysis of the effect of internal migration on subjective wellbeing for the population as a whole using the British Household Panel Study. They find a positive effect of a residential move on happiness, at least in the short term, and that this tends to be accompanied by a decline in happiness prior to migration. However, the effect of the move itself on wellbeing will depend on the event, or driver, or reason, for migration; indeed “[i]t would be desirable to investigate the happiness consequences of migration by migration motives” (Nowok et al., 2013, p. 999). The ageing literature tells us that declines in wellbeing in later life are expected, and are expected to be linked to events or changing circumstances associated with ageing, including loss of spouse, reduced social networks and illness or disability (Jivraj et al., 2014). Concurrently, migration literature has identified similar later life events as drivers of migration (Evandrou et al., 2010; Rees & Hardill, 2015). Thus, we can expect a decline in wellbeing in later life, and prior to migration, and can posit that the effect of migration on wellbeing in later life will depend on the reason for the move.

Migration in later life is associated with reasons other than those that dominate for the younger population, namely to do with work and study. Although retirement has been shown to be an important stimulus for migration, particularly in terms of counterurbanisation (Stockdale, 2016), residential decisions cannot be disassociated from other family, health or economic changes in later life. It has long been recognised in the ageing literature that the Third Age – that of early later life – can be a time of opportunities or constraints (Laslett, 1994). In relation to migration, a move might offer a means to maintain wellbeing in response to changing circumstances or may offer new life opportunities; or may be an undesired, involuntary response to deteriorating health, family or economic circumstances. The question we pose is whether migration's effect on wellbeing is different depending on the reasons for the move. Particularly, we address the following research questions: in later life, are there differences in trajectories of wellbeing for those who move and those who do not move? How do these trajectories vary for “voluntary” and “involuntary” movers? For later life movers, how does the move event affect trajectories (rates of decline) of wellbeing? How does the effect of the move event on wellbeing vary for “voluntary” and “involuntary” movers?

## 2 DATA AND METHODS

The analysis in this paper uses the ELSA, a survey on the health and circumstances of a representative sample of the population aged 50 and over living in private households in England. The initial ELSA sample in 2002 comprised a nationally representative sample of 11,391 people aged over 50 and living in private households and is drawn from the Health Survey for England. The baseline sample are re‐interviewed every two years and six waves of data spanning the period 2002 to 2012 are used in this paper. Methodological details on survey design and data collection are provided elsewhere (see Cheshire et al., 2012). The survey contains rich detail on the health, wellbeing and circumstances of older people, but crucially also detail on internal migration and the (self‐reported) reasons for residential moves.

Two different samples of ELSA are used within the two separate pieces of analysis reported in this paper. In order to compare wellbeing trajectories (or change in wellbeing, 2002–2012) for migrants and non‐migrants (research question 1), the first analysis uses the full ELSA sample. The second part of the analysis considers the impact of migration on wellbeing for a sub‐sample of individuals who moved at some point during the ELSA survey period (2002 to 2012; research question 2). We also include in our analysis the refreshment samples in wave 3 and wave 4. Thus for the second analysis our sample is 1,387 individuals.

Our measure of wellbeing is the CASP‐19 scale, which evaluates wellbeing (both hedonic and eudemonic) according to domains of Control, Autonomy, Self‐realisation and Pleasure as developed and validated specifically for older people by Wiggins et al. (2007). Summing scores across 19 Likert questions provides a continuous measure of wellbeing with higher scores indicating higher levels of wellbeing. A selection of questions across domains is illustrated in Table 1, with descriptive statistics illustrated in Table 2. One point to note from Table 2 is that the propensity to make voluntary or involuntary moves appears to be related to social class, health, living alone and tenure. Involuntary movers are more likely to be single, in poor health, in the poorest quintiles (of household wealth) and to rent their accommodation. One implication of this is that we must control for socio‐economic circumstances if we are to assess more accurately the influence of migration reason on wellbeing.

**Table 1:** Sample question statements from the Control, Autonomy, Self‐Realisation and Pleasure (CASP‐19) measure of wellbeing

**Control and autonomy**
My age prevents me from doing the things I would like to do.
I feel that what happens to me is out of my control.
I feel left out of things.
I can do the things I want to do.
I feel that I can please myself what I do.
Shortage of money stops me doing things I want to do.
**Self‐realisation**
I feel that life is full of opportunities.
I feel full of energy these days.
I feel that the future looks good for me.
**Pleasure**
I look forward to each day.
I feel that my life has meaning.
I enjoy the things that I do.

Respondents are asked to respond according to a Likert scale indicating the extent to which they agree/disagree with the statement. The CASP‐19 is the sum of the scores across the 19 items with a higher score indicating higher wellbeing.

**Table 2:** Baseline characteristics of English Longitudinal Study of Ageing respondents at wave 1 (core members) with a CASP‐19 score, 2002–2003

Variable	Full sample (core)^b^		Non‐movers (2002–2012)		Movers (2002–2012)		Involuntary movers (2002–2012)		Voluntary movers (2002–2012)	
	Mean or %	*N*	Mean or %	*N*	Mean or %	*N*	Mean or %	*N*	Mean or %	*N*
CASP score (mean)	42.5	9,300	42.4	7,879	42.8	852	40.9	305	43.9	547
Age (mean)	64.2	9,300	64.6	7,879	62.5	852	64.1	305	61.6	547
Male (%)	46.5	4,325	47.1	3,712	43.8	373	42.6	130	44.4	243
Wealth (%)^a^										
Quintile 1 (poorest)	16.8	1,534	16.1	1,249	19.3	163	24.7	74	16.4	89
Quintile 2	19.2	1,758	19.8	1,536	15.9	134	21.7	65	12.7	69
Quintile 3	20.6	1,882	20.9	1,623	17.4	147	17.7	53	17.3	94
Quintile 4	21.4	1,960	21.5	1,669	20.7	175	18.0	54	22.2	121
Quintile 5 (richest)	22.1	2,026	21.7	1,681	26.7	225	18.0	54	31.4	171
Has limiting long‐term illness (%)	32.8	3,052	32.7	2,575	31.0	264	43.6	133	24.0	131
Single (wave 2)	27.9	1,905	27.3	1,544	30.0	207	33.2	78	28.5	129
Tenure										
Owns home outright	56.6	5,261	58.3	4,590	46.8	399	42.6	130	49.2	269
Owns home with a mortgage	25.5	2,374	24.8	1,950	32.3	275	31.2	95	32.9	180
Rents	17.9	1,665	17.0	1,339	20.9	178	26.2	80	17.9	98
Economic activity										
Retired	49.3	4,564	50.3	3,952	41.5	352	46.2	141	38.9	211
Employed	34.5	3,190	33.4	2,623	41.4	351	32.5	99	46.4	252
Unemployed	1.0	91	0.9	73	1.4	12	1.3	4	1.5	8
Sick/disabled	5.8	539	5.7	447	5.9	50	8.5	26	4.4	24
Looking after home/family	9.5	877	9.6	755	9.8	83	11.5	35	8.8	48

a Wealth quintiles are based on the full ELSA sample who provided information to compute the wealth variable (*N* = 11,191) while we have CASP‐19 scores for a sub‐sample (*N* = 9,300). For this reason the split of the (full) sample across quintiles deviates slightly from 20% in each category. Our substantive results are not affected should we recalculate wealth quintiles for those who have a CASP‐19 score.

b Missing value for migration reason means that the counts of voluntary and involuntary moves don't sum to the full sample with a CASP‐19 score.

Internal migration is identified using a variable indicating whether an individual's address changed between survey waves. We determined the timing of move by drawing on a survey question on the year in which an individual moved to their current home. Based on year of move, we then constructed a variable indicating time to and from the move which varies from −10 (10 years before move) to 10 (10 years after move).

English Longitudinal Study of Ageing is a valuable source for research on internal migration in that it includes information on self‐reported reasons for moves. Respondents were able to select from a list of motivations for a residential move. We divided these into “voluntary” and “involuntary” reasons, as in Table 3, following the rule that if one involuntary reason was reported then the move was classed as involuntary. Inevitably there are issues around such classification. We might expect some bias in recollection of the reasons for a move after the move has occurred compared to before (post‐hoc rationalisation). And we might have followed other approaches in our definition of voluntary and involuntary mobility, particularly in the categorisation of moves in with, or to be near to, friends or family or those to care institutions. The principle we have followed in our categorisation with regard to these reasons for move is to assign those that imply increased social networks as voluntary.

**Table 3:** Categorisation of involuntary and voluntary residential moves as recorded in the English Longitudinal Study of Ageing

Involuntary (38% moves)	Voluntary (62% moves)
Job relocation (1.5%)	Start new job (1.9%)
Evicted, couldn't afford rent, or home repossessed (0.9%)	Moved to better area (14.5%)
Health reasons (20.0%)	Moved to more suitable home (42.2%)
Split from partner (4.1%)	Bought own home (1.7%)
Other financial reasons (9.5%)	Moved in with friends/family (3.4%)
Home poor condition or demolished (0.7%)	Moved to be nearer friends/family (17.6%)
Nursing home (0.5%)	Moved abroad (0.2%)
	Moved in with partner (3.2%)

In addition to our reason for migration variable, we include in our models a set of social and demographic control variables comprising age, sex, wealth, economic activity, self‐reported illness, tenure and cohabitation (living alone or with partner). Our measure of wealth includes both net financial and physical wealth (such as art and jewellery) and the net housing wealth for each household. We use a household measure of wealth because in the vast majority of cases older couples pool finances. Pension wealth is excluded from our analysis because it is particularly age dependent, declining throughout retirement. The sample characteristics stratified by migration status are provided in Table 1.

We fit two types of models; the first involves a multilevel growth curve (repeated observations nested within individuals) to examine how trajectories (or change) of wellbeing evolve between 2002 and 2012 for age cohorts (based on age at wave 1 – 2002). The approach employs a similar methodology to that used by Yang (2007) to model trajectories of depression, by Jivraj et al. (2014) to model wellbeing in later life and by Marshall et al. (2015) for frailty. A key advantage of using a multilevel approach is that it offers one way of dealing with the correlation in an individual's wellbeing over time and the technique is capable of handling unequal time spaces, missing data and the inclusion of time varying and between subject covariates that are either continuous or discrete measures (Raudenbush & Chan, 1992). We extend the analysis of wellbeing in later life reported by Jivraj et al. (2014) to include stratification by migration status (non‐movers, voluntary movers and involuntary movers) and we control for the set of socio‐demographic controls (age, sex, self‐reported illness, economic activity, tenure, economic activity, wealth). Essentially, the model produces a different trajectory for the mean level of wellbeing for each age cohort between 2002 and 2012. Further, it allows for the trajectories of (age cohort‐specific) wellbeing to vary across groups of non‐movers, voluntary movers and involuntary movers. Such cohort‐specific analysis is valuable to maximise the richness of the longitudinal ELSA data and to explore the possibility that the impact of migration on wellbeing might vary according to cohort. The socio‐demographic control variables protect against the possibility that differences in socio‐demographic profiles of non‐movers and movers (voluntary and involuntary) (see Table 2), rather than factors to do with internal migration, are driving differences in trajectories of wellbeing across migration/non‐migration groups. We explored the use of interactions between migration status and cohort and migration status and time, but as these did not attain statistical significance we do not include these interactions in the final models.

The second model is concerned with how wellbeing evolves as individuals experience a move (voluntary or involuntary). We model trajectories in the mean CASP score (wellbeing) using a repeated measures linear regression model fitted using the generalised estimating equations method which takes account of the autoregressive correlation structure (Lipsitz, Kim, & Zhao, 1994). We used a spline model with a knot at the point at which a move occurred and assess whether the pre‐move slope in mean CASP score with time differs to the slope of change after a move. In other words, we wish to determine whether the change in wellbeing after a move is different to before a move and whether this depends on the nature of the move (voluntary/involuntary). We interacted our pre and post‐move slope terms with a variable distinguishing whether a move is voluntary or involuntary. Again we control for a set of socio‐demographic variables (which take the value observed at the time point prior to migration) to guard against the possibility that the social and demographic profiles of the two migration groups drive any differences in trajectories observed. Similar modelling strategies have been used to model wellbeing and health outcomes through events such as migration (Nowok et al., 2013) and retirement (Marshall & Nazroo, 2015; Westerlund et al., 2009).

## 3 RESULTS

**Figure 1:**
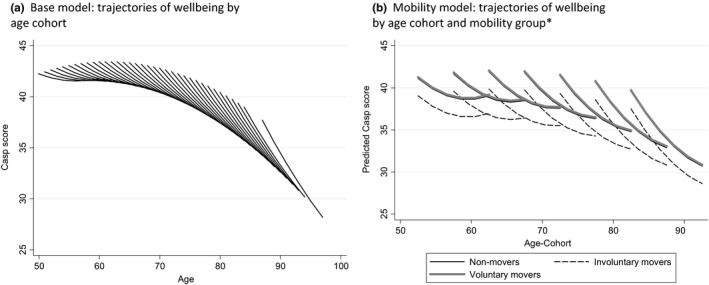
Trajectories (or change) in mean wellbeing (CASP‐19 score) between 2002 and 2012 by age‐cohorts. *The model wellbeing scores control for wealth quintiles and sex differences in mobility groups. For regression coefficients see supplementary material (Table S1). *Note*: In (a) cohorts are based on single years of age at 2002, whereas in (b) the inclusion of additional explanatory variables (e.g., reason for move) reduces sample sizes and requires the use of cohorts based on 5‐year age groups. (b) The modelled wellbeing scores control for gender, tenure, cohabitation, economic activity and self‐reported illness taking the reference category for each of these independent variables. Model trajectories for non‐movers and voluntary movers are almost identical and we found no evidence of any statistically significant difference in the level or gradient of wellbeing predicted.

**Figure 2:**
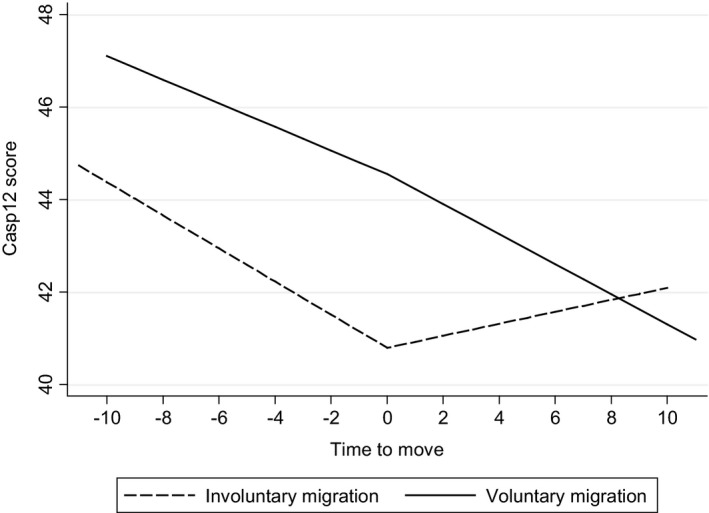
Trajectories (or change) in mean wellbeing (CASP‐19) scores before and after a residential move. Model wellbeing scores relate to age (in wave prior to move) of 50 and the reference category of all other independent variables. *Note*: For model coefficients and statistics see Table S2 (Mobility reasons model [controlled]). The model controls for economic activity, tenure, marital status, wealth, age and sex (for all these variables we use the value immediately prior to the move in our models).

Three key results emerge from our analyses (see Figures 1 and 2 and Tables S1 and S2). First, we observe lower levels of wellbeing for those who move for involuntary reasons compared with non‐movers and voluntary movers and this is consistent across age cohorts. Thus, internal migration in later life is selective in that wellbeing is lower for older people who move house for involuntary reasons. Second, for those people who moved for involuntary reasons, we observe a difference in the rate of change in wellbeing after a move compared with before. Specifically, the decline in wellbeing prior to a move is followed by a levelling off in the trajectory of wellbeing after a move. In other words, those in adverse circumstances with poor wellbeing who are in some way forced to migrate benefit from that internal migration in terms of their wellbeing not declining as much as expected without migration. Third, for those respondents who moved for voluntary reasons, the decline in wellbeing before and after migration is identical. In sum, internal migration is good for wellbeing in later life: older people who move for voluntary reasons maintain their relatively high levels of wellbeing even after controlling for correlates of wellbeing and those who move involuntarily experience a slower decline in wellbeing after compared with before a move.

In Figure 1a we see the evolution of wellbeing across age cohorts for the full ELSA sample. First, focusing on the level of wellbeing in 2002 across age cohorts (the start of each trajectory of wellbeing), we observe an inverted *U*‐shaped pattern with progressively higher level of wellbeing moving from the cohort aged 50 in 2002 to the cohort aged around 65 in 2002 followed by declines in wellbeing as one moves to earlier born cohorts (i.e., those aged 65 and over in 2002). The decline in wellbeing over time within age cohorts increases as one moves from later to earlier born cohorts and here we essentially replicate part of the cohort‐specific analysis of Jivraj et al. (2014), who discuss the social determinants of wellbeing in detail. An interesting point to note in relation to Figure 1a is the evidence for a difference in levels of wellbeing across cohorts when compared at the same age, such that earlier born cohorts tend to have higher wellbeing than later born cohorts, a similar finding to that reported for frailty by Marshall et al. (2015).

Figure 1b shows stratification by the migration variable (non‐mover, voluntary mover and involuntary mover) and Table S1 provides the coefficients that underpin the predicted CASP scores presented. While the trajectories of wellbeing are almost identical for voluntary movers and non‐movers (no statistically significant differences), we see clearly lower levels of wellbeing for involuntary movers that are statistically significant and are independent of the social and demographic control variables included in our model. The rate of change in wellbeing within age cohorts is comparable across mobility groups (we find no evidence of statistically significant differences).

Figure 2 shows the trajectory of wellbeing for all those individuals who moved during the ELSA study (2002–2012), with Table S2 illustrating the model coefficients. For voluntary movers there is no evidence for difference in the slope of wellbeing before and after a move, whereas for those who moved for reasons classed as “involuntary,” after a move we observe a levelling off in the decline in wellbeing observed before the move. This difference in slopes of mean wellbeing either side of a move is statistically significant (*p* = 0.04, see Table S2) and holds after controlling for a set of socio‐demographic controls. So, those in adverse circumstances with poor wellbeing who are in some way forced to migrate benefit from that internal migration in terms of their wellbeing not declining as much as expected without migration.

## 4 DISCUSSION AND CONCLUSIONS

We have addressed in this paper the questions of the differences in wellbeing in later life for those who move house and those who do not, and the effect of the move event on wellbeing. In both cases, we have distinguished between moves for voluntary reasons and moves for involuntary reasons. This paper thus adds to literature on non‐economic effects of internal migration, and in doing so contributes to the relatively neglected field of internal migration in later life, with implications for understandings of how migration is selective and may thus affect ageing inequalities. We have employed longitudinal analysis of the ELSA to allow us to identify trends in wellbeing, and how these vary across cohorts and before and after a residential move.

Our key result is that migration in later life is good for wellbeing: for those who move voluntarily, it maintains high levels of wellbeing (independently of other socio‐economic and demographic correlates of wellbeing) and for those whose moves are involuntary it stabilises the decline in wellbeing associated with ageing. More precisely, our analyses found that voluntary movers have high levels of wellbeing, comparable with those who don't move (stayers), which in turn are higher than for those who move for involuntary reasons. The trajectory of wellbeing for those who move involuntarily is improved compared with the period prior to the move; there is no such change in wellbeing trajectory after a move compared with before for those who move for voluntary reasons, though their wellbeing levels remain above those of people who move for involuntary reasons at all points. The differences in levels of wellbeing trajectories and the effect of an involuntary move on the trajectory of wellbeing after a move (compared with before) are statistically significant and remain after controlling for the characteristics of the groups of voluntary movers/involuntary movers/stayers, including in terms of gender and wealth.

There are a number of implications of our study, for methods, concepts of internal migration, studies of ageing inequalities and policy. On methods, this work supports others in demonstrating that large‐scale, longitudinal data with migration and reason for move information provide a rich opportunity for better understanding of migration as a process of selection and social inequalities. ELSA has been shown to be an excellent resource in this respect and more use could be made of this survey, and its sister studies in other countries.

For concepts of (internal) migration, our work reinforces the point often made – but rarely operationalised in quantitative studies – that it is crucial to know reason for move in order to understand migration experiences and consequences. Moreover, the findings offer an alternative conceptualisation of the impact of involuntary moves. In migration literature (aside from forced migration literature), involuntary moves have been most studied in relation to tied migrants, those who move for reasons related to the partner's work. Tied migrants (usually female) have been shown to experience economic disadvantage after migration. The divergence in our findings and Nowok et al. (2013) also illustrates the importance of focusing on later life, and how not only drivers of internal migration but their consequences may differ from at other life stages. Finally, this study has demonstrated how internal migration studies can contribute to debates about inequalities by paying greater attention to drivers of migration, and how they represent inequalities in choice and constraint, across the lifecourse.

For scholarship on ageing inequalities, this paper illustrates the need to take into account residential experience and change. Wealth inequalities in later life are part of the explanation of differential associations between migration and wellbeing (poor but not rich tend to be “involuntary” movers), but aside from the wealth of individuals, whether a move is voluntary or involuntary is independently associated with wellbeing.

For policy and practice, including place‐based initiatives such as the WHO Age Friendly Cities movement, our analysis offers a useful pointer for maximising wellbeing in later life. Policies that enable migration of older people may be valuable in maintaining wellbeing and, for those facing adverse circumstances, may stabilise decline in wellbeing, thus reducing inequalities in wellbeing in later life.

Of course, there is much that we have not done in this paper that could usefully augment understandings of whether migration is good for wellbeing and implications for inequalities in ageing. In particular, we do not capture anything about the household or family links of movers or stayers, which are likely to be crucial for understanding drivers and impacts of internal migration in later life (van der Pers et al., 2015). In this respect there are undoubtedly gender differences to explore. It would be useful also to be able to compare voluntary and involuntary movers to voluntary and involuntary stayers. This would enable, for example, assessment of the extent to which there is unfulfilled moving desire (Coulter, 2013) among older people and how enabling migration may potentially enhance wellbeing. One important limitation of this study is attrition of sample members, which is more common for those with poorer wellbeing/health and in the lower social classes. We cannot entirely rule out the possibility that the levelling off in the trajectory of wellbeing after an involuntary move, compared with the trajectory before the move, is not driven to some extent by attrition of those with low levels of wellbeing. We guard against this to some extent in that replication of our analysis using the ELSA wave 6 longitudinal weights, which account for attrition (but lead to a much smaller sample size requiring observations at each wave), does not alter our substantive findings. Similarly, we must acknowledge that ELSA is a household survey, excluding those in care institutions for whom different drivers of migration and relationships with wellbeing are likely. Place is entirely absent from our study; where people move to and from and how this relates to family and social networks, services and suchlike is undoubtedly important for understanding the association between moving and wellbeing. For example, the body of research that has demonstrated migration to be health‐selective, with the effect of increasing spatial inequalities in health outcomes, might be further developed through stratification according to reason for migration (Norman & Boyle, 2014; Norman et al., 2005). Additionally, this paper would ideally be developed with greater attention to reason for move beyond the rather crude categorisation of “voluntary” and “involuntary” that we have employed here. This in itself could constitute a programme of work, qualitative and quantitative. We hope that further research will address these deficiencies. Given the declines in residential mobility over time, including for older age groups (Champion & Shuttleworth, 2016), combined with our finding of the positive effects of migration for wellbeing, particularly for those facing adverse circumstances, the question of who gets to move becomes increasingly salient for those interested in challenging social inequalities.

## Supporting information


**Table S1**. Model statistics: trajectories (change) in wellbeing (CASP19) in later life, for non‐migrants, voluntary movers and involuntary movers (the coefficients are used to generate the predicted wellbeing trajectories displayed in Figure 1).
**Table S2**. Model statistics: trajectories (change) in wellbeing (CASP19) through a residential move (the coefficients are used to generate the predicted wellbeing trajectories displayed in Figure 2).
