# Supplementary material for: Is migration in later life good for wellbeing? A longitudinal study of ageing and selectivity of internal migration
Source: Area (Oxf). 2018 May 17;50(4):492–500. doi: 10.1111/area.12428 (PMC6282955; doi:10.1111/area.12428)
Supplement: Supplementary file 1 — Table S1. Model statistics: trajectories (change) in wellbeing (CASP19) in later life, for non‐migrants, voluntary movers and involuntary movers (the coefficients are used to generate the predicted wellbeing trajectories displayed in Figure 1). Table S2. Model statistics: trajectories (change) in wellbeing (CASP19) through a residential move (the coefficients are used to generate the predicted wellbeing trajectories displayed in Figure 2). [file AREA-50-492-s001.docx]

**Supplementary material**

|  |  | **Model 1 - empty model** | | **Model 2 - mobility reason* (uncontrolled)** | | **Model 3 - mobility reason* (controlled)** | |
| --- | --- | --- | --- | --- | --- | --- | --- |
|  |  | Coef | p>\|z\| | Coef | p>\|z\| | Coef | p>\|z\| |
| Intercept | | 8.04 | 0.03 | 3.26 | 0.45 | 14.67 | 0.002 |
| Cohort | | 1.14 | <0.0001 | 1.28 | <0.0001 | 0.88 | <0.0001 |
| Cohort^2^ | | -0.01 | <0.0001 | -0.01 | <0.0001 | -0.01 | <0.0001 |
| Wave | | 1.86 | <0.0001 | 1.84 | <0.0001 | 0.57 | 0.018 |
| Wave^2^ | | 0.07 | <0.0001 | 0.07 | <0.0001 | 0.20 | <0.0001 |
| Wave*Cohort | | -0.05 | <0.0001 | -0.05 | <0.0001 | -0.05 | <0.0001 |
| Involuntary migration adjustment to intercept | |  |  | -2.42 | <0.0001 | -2.06 | <0.0001 |
| Voluntary migration adjustment to intercept | |  |  | 0.87 | 0.01 | 0.16 | 0.594 |
| Female | |  |  |  |  | 0.60 | <0.0001 |
| Wealth (reference category is the poorest quintile) | |  |  |  |  |  |  |
| 2nd poorest quintile | |  |  |  |  | 2.37 | <0.0001 |
| Middle wealth quintile | |  |  |  |  | 3.32 | <0.0001 |
| 2nd richest quintile | |  |  |  |  | 4.46 | <0.0001 |
| Richest quintile | |  |  |  |  | 6.20 | <0.0001 |
| Tenure (own outright is the reference) | |  |  |  |  |  |  |
| Own with mortgage |  |  |  |  |  | -0.52 | <0.0001 |
| Rent |  |  |  |  |  | -0.20 | 0.445 |
| Economic activity (Retired is the reference) | |  |  |  |  |  |  |
| Employed |  |  |  |  |  | 0.02 | 0.909 |
| Unemployed |  |  |  |  |  | -1.46 | <0.0001 |
| Sick/disabled |  |  |  |  |  | -3.59 | <0.0001 |
| Looking after home/family | |  |  |  |  | -0.32 | 0.033 |
| Living with partner (reference is living with no partner) | | |  |  |  | 0.16 | 0.299 |
| Has a limiting long term illness (reference is no illness) | | |  |  |  | -2.57 | <0.0001 |
| **Random effects (95% c.i.)** | |  |  |  |  |  |  |
| **Level 2 (across persons)** | |  |  |  |  |  |  |
| Var(constant) | | 62.5 (61.1-65.9) | | 63.4 (60.9-65.9) | | 53.5 (50.3-56.8) | |
| Cov(constant,wave) | | -2.8 (-3.2--2.3) | | -2.8 (-3.2--2.4) | | -3.2 (-3.8 - -2.6) | |
| Var(wave) | | 1.1 (1.1-1.2) | | 1.1 (1.0-1.2) | | 1.0 (0.9 - 1.1) | |
| **Level 1 (within person)** | |  |  |  |  |  |  |
| Var(Constant) | | 17.4 (17.1-17.8) | | 17.4 (17.1-17.8) | | 16.5 (16.1-16.9) | |
| **Model fit** | |  |  |  |  |  |  |
| Log-likelihood | | -119,875.85 | | -111,689.83 | | -80,747.45 | |

**Table S1**: Model statistics: Trajectories (change) in wellbeing (CASP19) in later life, for non-migrants, voluntary movers and involuntary movers (the coefficients are used to generate the predicted wellbeing trajectories displayed in Figure 1)

*Mobility reason distinguishes whether a move was voluntary or involuntary

|  | Model 1 - empty model | | Model 2: Migration reason (uncontrolled) | | Model 3 - Migration reason: controlled) | |
| --- | --- | --- | --- | --- | --- | --- |
|  | Coef. | p>\|z\| | Coef. | p>\|z\| | Coef. | p>\|z\| |
| Constant | 41.81 | <0.001 | 38.32 | <0.001 | 43.46 | <0.001 |
| Slope | -0.2 | 0.29 | -0.27 | 0.02 | -0.36 | 0.002 |
| Post-move slope adjustment | 0.08 | <0.001 | 0.42 | 0.04 | 0.49 | 0.043 |
| Voluntary move adjustment to constant |  |  | 4.65 | <0.001 | 3.75 | <0.001 |
| Voluntary move adjustment to slope |  |  | 0.05 | 0.71 | 0.10 | 0.468 |
| Voluntary move (additional) post-move slope adjustment |  |  | -0.54 | 0.03 | -0.56 | 0.047 |
| Age |  |  |  |  | -0.11 | 0.038 |
| Female |  |  |  |  | 1.31 | 0.099 |
| Wealth (reference category is the poorest quintile) |  |  |  |  |  |  |
| 2nd poorest quintile |  |  |  |  | -0.69 | 0.683 |
| Middle wealth quintile |  |  |  |  | -1.37 | 0.492 |
| 2nd richest quintile |  |  |  |  | -0.21 | 0.912 |
| Richest quintile |  |  |  |  | 0.77 | 0.68 |
| Living with partner (reference is living alone) |  |  |  |  | 2.60 | 0.011 |
| Tenure (reference is own outright) |  |  |  |  |  | |
| Own with mortgage |  |  |  |  | 0.68 | 0.468 |
| Rent |  |  |  |  | -1.93 | 0.222 |
| Economic activity (reference is retired) |  |  |  |  |  |  |
| Employed |  |  |  |  | 1.26 | 0.166 |
| Unemployed |  |  |  |  | -2.64 | 0.349 |
| Sick/disabled |  |  |  |  | -8.39 | <0.001 |
| Looking after home and family |  |  |  |  | -1.35 | 0.439 |
| **Model statistics** |  |  |  |  |  |  |
| Wald Chi-squared statistic | 28.4 | | 70.91 | | 140.10 | |
| Prob>chi-squared statistic | <0.001 | | <0.001 | | <0.001 | |
| Number of individuals | 1204 | | 585 | | 362 | |
| Number of observations | 4997 | | 2624 | | 1883 | |
| Average number of observations per individual | 4.2 | | 4.5 | | 5.2 | |

**Table S2**: Model statistics: trajectories (change) in wellbeing (CASP19) through a residential move (the coefficients are used to generate the predicted wellbeing trajectories displayed in Figure 2)

*Mobility reason distinguishes whether a move was voluntary or involuntary
